# ﻿Revision of the banding sequence pool and new data on chromosomal polymorphism in natural populations of *Chironomusagilis* Shobanov et Djomin, 1988 (Diptera, Chironomidae)

**DOI:** 10.3897/CompCytogen.v15.i4.76761

**Published:** 2021-12-17

**Authors:** Veronika V. Golygina, Oksana V. Ermolaeva

**Affiliations:** 1 The Federal Research Center Institute of Cytology and Genetics of Siberian Branch of the Russian Academy of Science, Prospect academika Lavrentieva 10, Novosibirsk, 630090 Russia Novosibirsk State University Novosibirsk Russia; 2 Novosibirsk State University, ul. Pirogova, 2, Novosibirsk, 630090 Russia The Federal Research Center Institute of Cytology and Genetics of Siberian Branch of the Russian Academy of Science Novosibirsk Russia

**Keywords:** Banding sequence, *Ch.plumosus* group, inversion, karyotype, karyological analysis, polythene chromosome, sibling species

## Abstract

Quantitative and qualitative analysis of chromosomal polymorphism in 19 natural populations of *Ch.agilis* had been performed. Most studied populations showed a medium level of chromosomal polymorphism: on average 45±3.0% of specimens are heterozygotes with 0.52±0.01 heterozygotic inversion per larvae. Besides inversions, B-chromosomes were found in two populations. The total number of banding sequences found in banding sequence pool of *Ch.agilis* is 16. Three banding sequences – p’agiB3, p’agiD3, p’agiF3 – are described for the first time.

## ﻿Introduction

*Chironomusagilis* Shobanov et Djomin, 1988 belongs to the *Ch.plumosus* group of sibling species. This group presents a good model for studies of the evolution of the karyotype during speciation as well as chromosomal polymorphism in natural populations. Three species with the widest ranges – *Ch.plumosus* (Linnaeus 1758), *Ch.entis* Schobanov, 1989 and *Ch.balatonicus* Dévai, Wülker et Scholl, 1983 – have been studied most extensively. At the same time, only a few populations were studied karyologically for other species from the group. In case of *Ch.agilis* the data on chromosomal polymorphism were published only for two populations – one from Eastern Europe (Rybinskoe reservoir) and one from Siberia (Berdsky pond) – where only 9 banding sequences had been found in total (Schobanov and Djomin 1988; Kerkis et al. 1989). Two other studies where data on chromosomal polymorphism have been published cannot be taken into account as the karyotypes shown on photos designated as *Ch.agilis* actually belong to different species (Michailova et al. 2002; Krastanov and Michailova 2008). Thus, based on the known data the species could be considered as having a low level of polymorphism. In the book of Kiknadze and coauthors (2016) four new banding sequences were described, which brought the total number of banding sequences known for *Ch.agilis* to 13, but no new data on quantitative characteristics were published as it was not the purpose of that work.

In this paper we present data on chromosomal polymorphism in 19 natural populations of *Ch.agilis* from Eastern Europe, Siberia and the Far East.

## ﻿Material and methods

The VI instar larvae from 19 natural populations of Eastern Europe, Siberia and the Far East were used for slide preparations of polytene chromosomes. The data on collection sites and larvae studied are presented in Table 1. In cases when larvae were obtained several times over years from the same collection site, data on all probes were combined as statistical analysis (Fisher criteria, Plokhinsky 1967) had shown that there were no significant differences in frequencies of banding sequences between probes.

**Table 1.** Collection sites.

Collection place	Abbreviation	Collection date	Geographic coordinates	Number of larvae
**Yaroslavl region**				
Rybinsk Reservoir	YAR-RY	13.05.1988 11.07.1988 01.08.1988	58°11'59.4"N, 38°24'59.5"E	4
**Novosibirsk region**				
Berdsky Pond, the Shadrikha Rivulet, Berdsk	NSK-BE	27.05.1985 18.06.1986 02.06.1987	54°43'60.0"N, 83°07'43.4"E	45
The Eltsovka River, Novosibirsk	NSK-EL	14.05.2001 16.05.2001	54°53'22.6"N, 83°05'27.5"E	4
Kainka Lake, Kainskaya Zaimka settlement	NSK-KA	20.09.1989 27.04.1991	54°52'13.7"N, 83°08'09.7"E	34
The Shadrikha River, mouth	NSK-SH	07.05.2008 12.05.2011 11.05.2012 05.05.2014 04.05.2016 05.05.2017	54°46'41.1"N, 83°10'14.0"E	259
Bol’shaya Protoka Lake, Rechport, Novosibirsk	NSK-2R	13.05.2005	54°56'06.5"N, 83°03'46.0"E	14
Pond on the Shipunikha River, Iskitim	NSK-SP	07.05.2015	54°34'13.6"N, 83°20'42.6"E	1
Pond on the Koynikha River, Linevo settlement	NSK-LI	18.05.2006	54°27'45.5"N, 83°20'49.5"E	32
Pond on the Chernodyrikha River, Ryabchinka village	NSK-CH	16.05.2006	54°35'59.9"N, 83°07'57.8"E	2
Pond on the Sarbayan River, Uchastok-Balta village	NSK-SA	16.05.2002	55°24'42.3"N, 83°56'20.1"E	5
Pond on the Ora River, Sokur settlement	NSK-OR	17.05.2002 12.05.2006	55°12'58.8"N, 83°18'06.9"E	52
Pond on the Tars’ma River, Yurti settlement	NSK-YU	14.05.2002 22.05.2004	54°51'04.7"N, 84°51'04.9"E	151
Pond on the Tars’ma River, Stepnogutovo settlement	NSK-ST	12.05.2011 12.05.2016	54°51'08.6"N, 84°57'31.6"E	54
**Kemerovo region**				
Tanaevo Lake, Zhuravlevo settlement	KEM-TA	14.05.2002	54°46'35.0"N, 85°02'52.4"E	1
**Altai territory**				
Gilovskoye Reservoir	ALT-GI	15.05.2003	51°04'14.3"N, 81°59'57.7"E	1
Travinayoe Lake, Oskolkovo settlement	ALT-TR	08.05.1994	52°19'11.9"N, 83°11'24.2"E	3
**Khabarovsk territory**				
The Amur River, Khabarovsk	KHA-AM	21.06.1987	48°24'56.5"N, 135°05'39.4"E	2
Evoron Lake	KHA-EV	17.07.2006	51°23'02.9"N, 136°27'55.8"E	36
**Sakha Republic (Yakutiya)**				
Solyonoe Lake, Yakutsk vicinity	YAK-SO	05.09.1987	61°57'51.3"N, 129°37'01.1"E	2

The larvae were fixated with 3:1 *v/v* of 96% ethanol and glacial acetic acid and stored at –20 °C. Polytene chromosome squashes were prepared by a routine aceto-orcein method (Keyl and Keyl 1959; Kiknadze et al. 1991). Chromosomal mapping of arms A, C, D, E and F was done using mapping system created by Keyl (1962) and Devai et al. (1989), with *Ch.piger* Strenzke, 1959 as the standard karyotype. Mapping of arm B was done according to Maximova mapping system (Maximova 1976) improved by Schobanov (1994), with *Ch.plumosus* chromosomes as the standard.

Each banding sequence is given a short designation as follows: three-letter abbreviation of the species name (agi for *Ch.agilis*) followed by the name of the arm and the serial number of banding sequence in this arm (according to the order of its discovery), and prefixed by a letter indicating its geographical distribution in the genus *Chironomus* (p’ for Palearctic sequences or h’ for Holarctic sequences). Thus, for example, h’agiE1 means that while Ch.agilis itself is a Palearctic species, this banding sequences is identical to banding sequences of some other species and was found in Nearctic populations of those species, thus have a Holarctic distribution

The statistical analysis and phylogenetic tree construction was done using programs PHYLIP (https://evolution.genetics.washington.edu/phylip.html) and MEGA11.0.8 (https://www.megasoftware.net). Genetic distances between populations were calculated using Nei criteria (Nei 1972). The neighbor-joining method was used for construction of the phylogenetic tree between populations.

The equipment of the Centre of Microscopical analysis of biological objects SB RAS in the Institute of Cytology and Genetics (Novosibirsk) was used for this work: microscope “Axioskop” 2 Plus, CCD-camera AxioCam HRc, software package AxioVision 4 (Zeiss, Germany).

## ﻿Results and discussion

**Figure 1.**
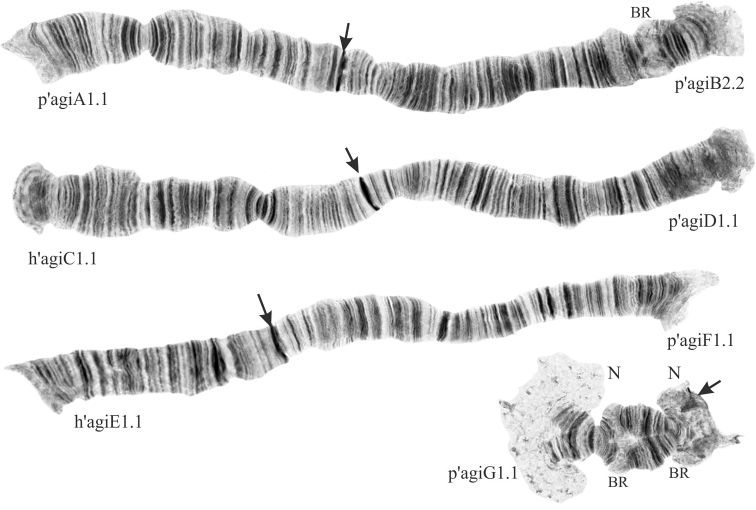
Karyotype of *Chironomusagilis*; p’agiA1.1, p’agiB2.2 etc. – genotypic combinations of banding sequences; BR – Balbiani ring, N – nucleolus. Arrows indicate centromeric regions.

The species *Ch.agilis* belongs to “thummi” cytocomplex with haploid number of chromosomes n=4 and arm combination AB CD EF G. The chromosomes I (AB) and II (CD) are metacentric, III (EF) is submetacentric, and IV (G) is telocentric (Fig. 1). There are two nucleoli in *Ch.agilis* karyotype; both are situated on the arm G – one on the centromeric end of the arm, the other on the opposite end near the telomere. Such location of nucleoli is the distinctive feature of *Ch.agilis* karyotype that allows to easily differentiate it from karyotypes of other *Chironomus* species. The homologues of arm G are paired but often are unconjugated at the ends in nucleolus organiser regions. The centromeric regions are distinct and easily identifiable on all chromosomes with the exception of the arm G where the centromere sometime can be masked by the nucleolus. There are three Balbiani Rings (BR) in the karyotype of *Ch.agilis*: two are situated on the arm G (usually only the one in the center of the arm is visible as the other one is often masked by the nucleolus), and the third one – on the arm B. Unlike in many other species from the genus *Chironomus*, BR on the arm B is usually well developed and easily identifiable in *Ch.agilis*.

**Figure 2.**
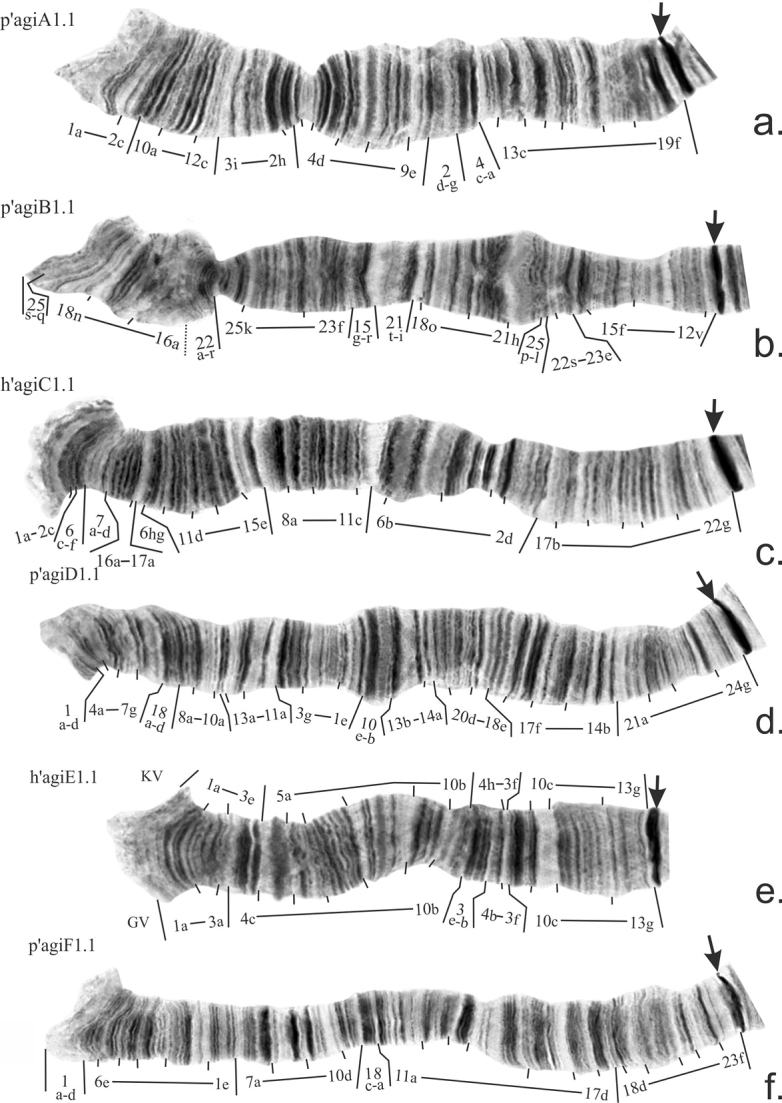
Mapping of main banding sequences in arms **a–f** of *Ch.agilis*. Arrows indicate centromeric regions. KV – version of mapping in arm E according to Keyl (1962), GV – version of mapping in arm E according to Golygina and Kiknadze (2018).

The revision of mapping of main banding sequences in arms A, B, C, D, E and F was presented by Golygina and Kiknadze previously (2008, 2012, 2018). Revised mapping of these banding sequences is shown on Fig. 2. For arm E two versions of mapping are presented (Fig. 2e). First is done according to how *Ch.agilis* banding sequence should be mapped if mapping of *Ch.plumosus* – reference species for mapping of all *Ch.plumosus* group sibling species – made by Keyl (1962) is considered to be correct (marked as KV). The second one is done according to revised mapping of *Ch.plumosus* made by Golygina and Kiknadze (2018) (marked as GV).

Inversion polymorphisms were found in all chromosome arms except G, but only arms B and F show a noticeably high level of polymorphism throughout different populations. Only rare or unique inversion banding sequences were found in arms A, C, D, and E. In total, 16 banding sequences were present in the studied populations.

**Figure 3.**
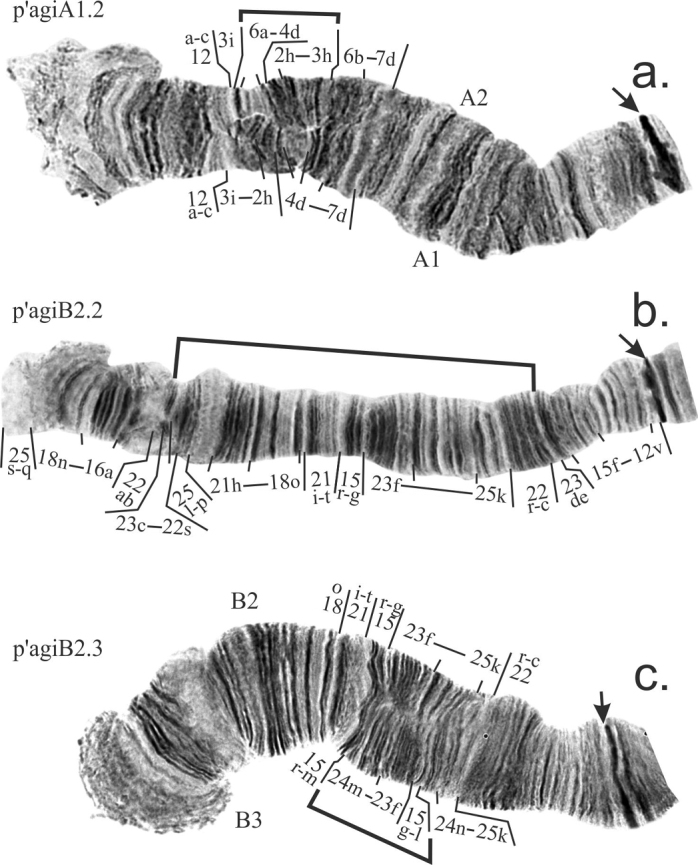
Inversions in arms A (**a**) and B (**b, c**) found in populations of *Ch.agilis.* Arrows indicate centromeric regions. Brackets show regions of inversions.

**Arm A** was monomorphic in all populations with the exception of the Siberian population from the pond on the Eltsovka river where banding sequence p’agiA2 was found in a heterozygous state in a single larvae (Table 2, 3). It differs from the main banding sequence p’agiA1 by the simple short paracentric inversion (Fig. 3a).

**Table 2.** Frequencies of genotypic combinations of banding sequences in populations of *Ch.agilis*.

Genotypic combination	Eastern Europe		Siberia																the Far East	
	YAR-RY†		NSK-BE	NSK-EL	NSK-KA	NSK-SH	NSK-2R	NSK-SP	NSK-LI	NSK-CH	NSK-SA	NSK-OR	NSK-YU	NSK-ST	KEM-TA	ALT-GI	ALT-TR	YAK-SO	KHA-EV	KHA-AM
	100‡§	4	45	4	34	259	14	1	32	2	5	52	151	54	1	1	3	2	36	2
p’agiA1.1	**1**	1	**1**	0.750	**1**	**1**	**1**	1	**1**	1	1	**1**	**1**	**1**	1	1	1	1	**1**	1
p’agiA1.2	**0**	0	**0**	0.250	**0**	**0**	**0**	0	**0**	0	0	**0**	**0**	**0**	0	0	0	0	**0**	0
p’agiB1.1	**1**	1	**0.044**	0	**0.029**	**0.042**	**0**	0	**0.031**	1	0	**0.019**	**0.007**	**0.056**	0	0	0	0.500	**0**	0.500
p’agiB2.2	**0**	0	**0.689**	1	**0.471**	**0.456**	**0.571**	1	**0.313**	0	1	**0.712**	**0.702**	**0.481**	1	1	0.667	0	**1**	0.500
p’agiB1.2	**0**	0	**0.267**	0	**0.500**	**0.498**	**0.429**	0	**0.656**	0	0	**0.269**	**0.291**	**0.463**	0	0	0.333	0.500	**0**	0
p’agiB2.3	**0**	0	**0**	0	**0**	**0.004**	**0**	0	**0**	0	0	**0**	**0**	**0**	0	0	0	0	**0**	0
h’agiC1.1	**1**	1	**1**	1	**1**	**1**	**1**	1	**1**	1	1	**1**	**1**	**1**	1	1	0.333	1	**1**	1
h’agiC1.p’agiC2	**0**	0	**0**	0	**0**	**0**	**0**	0	**0**	0	0	**0**	**0**	**0**	0	0	0.667	0	**0**	0
p’agiD1.1	**1**	1	**1**	1	**1**	**0.996**	**1**	1	**1**	1	1	**1**	**1**	**0.981**	1	1	1	1	**0.972**	1
p’agiD1.2	**0**	0	**0**	0	**0**	**0**	**0**	0	**0**	0	0	**0**	**0**	**0**	0	0	0	0	**0.028**	0
p’agiD1.3	**0**	0	**0**	0	**0**	**0.004**	**0**	0	**0**	0	0	**0**	**0**	**0.019**	0	0	0	0	**0**	0
h’agiE1.1	**1**	1	**1**	1	**1**	**0.996**	**1**	1	**1**	1	1	**1**	**1**	**1**	1	1	1	1	**1**	1
h’agiF1.p’agiE2	**0**	0	**0**	0	**0**	**0.004**	**0**	0	**0**	0	0	**0**	**0**	**0**	0	0	0	0	**0**	0
p’agiF1.1	**1**	1	**0.800**	0.750	**0.824**	**0.822**	**0.857**	1	**0.656**	1	1	**0.673**	**0.709**	**0.778**	0	0	0	0	**1**	1
p’agiF2.2	**0**	0	**0**	0	**0**	**0**	**0**	0	**0.063**	0	0	**0**	**0.007**	**0**	0	0	0	0	**0**	0
p’agiF1.2	**0**	0	**0.200**	0.250	**0.176**	**0.174**	**0.143**	0	**0.281**	0	0	**0.327**	**0.284**	**0.222**	1	1	1	1	**0**	0
p’agiF1.3	**0**	0	**0**	0	**0**	**0.004**	**0**	0	**0**	0	0	**0**	**0**	**0**	0	0	0	0	**0**	0
p’agiG1.1	**1**	1	**1**	1	**1**	**1**	**1**	1	**1**	1	1	**1**	**1**	**1**	1	1	1	1	**1**	1
B-chromosome	**0**	0	**0**	0	**0**	**0.050**	**0**	0	**0**	0	0	**0.038**	**0**	**0**	0	0	0	0	**0**	0
Number of banding sequences	**7**	7	**9**	9	**9**	**12**	**9**	7	**9**	7	7	**9**	**9**	**10**	7	7	9	8	**8**	8
Number of genotypic combinations of banding sequences	**7**	7	**10**	9	**10**	**14**	**9**	7	**11**	7	7	**10**	**11**	**11**	7	7	9	8	**8**	8
% of heterozygous larvae	**0**	0	**44.4**	50.0	**58.8**	**60.6**	**50.0**	0	**73.1**	0	0	**50.0**	**52.3**	**59.3**	0	0	66.7	50.0	**2.8**	0
Number of heterozygous inversions per larvae	**0**	0	**0.467**	0.500	**0.676**	**0.684**	**0.572**	0	**0.937**	0	0	**0.596**	**0.576**	**0.685**	0	0	1.0	0.500	**0.028**	0

† – populations highlighted with bold were used for quantitative analysis of chromosomal polymorphism; ‡ – number of larvae studied; § – the data were published by Shobanov and Djomin (1988).

**Table 3.** Frequencies of banding sequences in populations of *Ch.agilis*.|

Banding sequnce	Europe	Siberia								the Far East
	YAR-RY	NSK-BE	NSK-KA	NSK-SH	NSK-2R	NSK-LI	NSK-OR	NSK-YU	NSK-ST	KHA-EV
	100¶	45	34	259	14	32	52	151	54	36
p’agiA1	1	1	1	1	1	1	1	1	1	1
p’agiA2	0	0	0	0	0	0	0	0	0	0
p’agiB1	1	0.178	0.279	0.291	0.214	0.359	0.154	0.152	0.288	0
p’agiB2	0	0.822	0.721	0.707	0.786	0.641	0.846	0.848	0.712	1
p’agiB3	0	0	0	0.002	0	0	0	0	0	0
h’agiC1	1	1	1	1	1	1	1	1	1	1
p’agiC2	0	0	0	0	0	0	0	0	0	0
p’agiD1	1	1	1	0.998	1	1	1	1	0.991	0.986
p’agiD2	0	0	0	0	0	0	0	0	0	0.014
p’agiD3	0	0	0	0.002	0	0	0	0	0.009	0
h’agiE1	1	1	1	0.996	1	1	1	1	1	1
p’agiE2	0	0	0	0.004	0	0	0	0	0	0
p’agiF1	1	0.900	0.912	0.911	0.929	0.797	0.837	0.851	0.889	1
p’agiF2	0	0.100	0.088	0.087	0.071	0.203	0.163	0.149	0.111	0
p’agiF3	0	0	0	0.002	0	0	0	0	0	0
p’agiG1	1	1	1	1	1	1	1	1	1	1

| – only populations with enough larva for quantitative analysis (more than 10 specimens) are included into this table. ¶ – number of larvae studied.

p’agiA1 1a-2c 10a-12c 3i-2h 4d-9e 2d-g 4c-a 13a-19f C

p’agiA2 1a-2c 10a-12c 3i 6a-4d 2h-3h 6b-9e 2d-g 4c-a 13a-19f C

**Arm B** is polymorphic. The standard banding sequence p’agiB1 was found in most populations, but shows high frequency of occurrence only in the western part of the species range – in the population from Rybinsk Reservoir (Table 2, 3).

p’agiB1 25s-q 18n-16a 22a-r 25k-23f 15g-r 21t-i 18o-21h 25p-l 22s-23e 15f-12v C

In all studied populations from Siberia and Far East the alternative banding sequence p’agiB2 was dominant, although only in one population – from Evoron lake – its frequency reached 100% (Table 2, 3). This banding sequence differs from p’agiB1 by the large simple inversion (Fig. 3b).

p’agiB2 25s-q 18n-16a 22ab 23c-22s 25l-p 21h-18o 21i-t 15r-g 23f-25k 22r-c 23de 15f-12v C

The banding sequence p’agiB3 is a short simple inverstion in the middle of the arm that is originated from p’agiB2 and was found only once in the population from the pond on the Shadrikha river (Table 2, 3, Fig. 3c). The banding sequence p’agiB3 is new for the species and described for the first time.

p’agiB3 25s-q 18n-16a 22ab 23c-22s 25l-p 21h-18o 21i-t 15r-m 24m-23f 15g-l 24n-25k 22r-c 23de 15f-12v C

**Figure 4.**
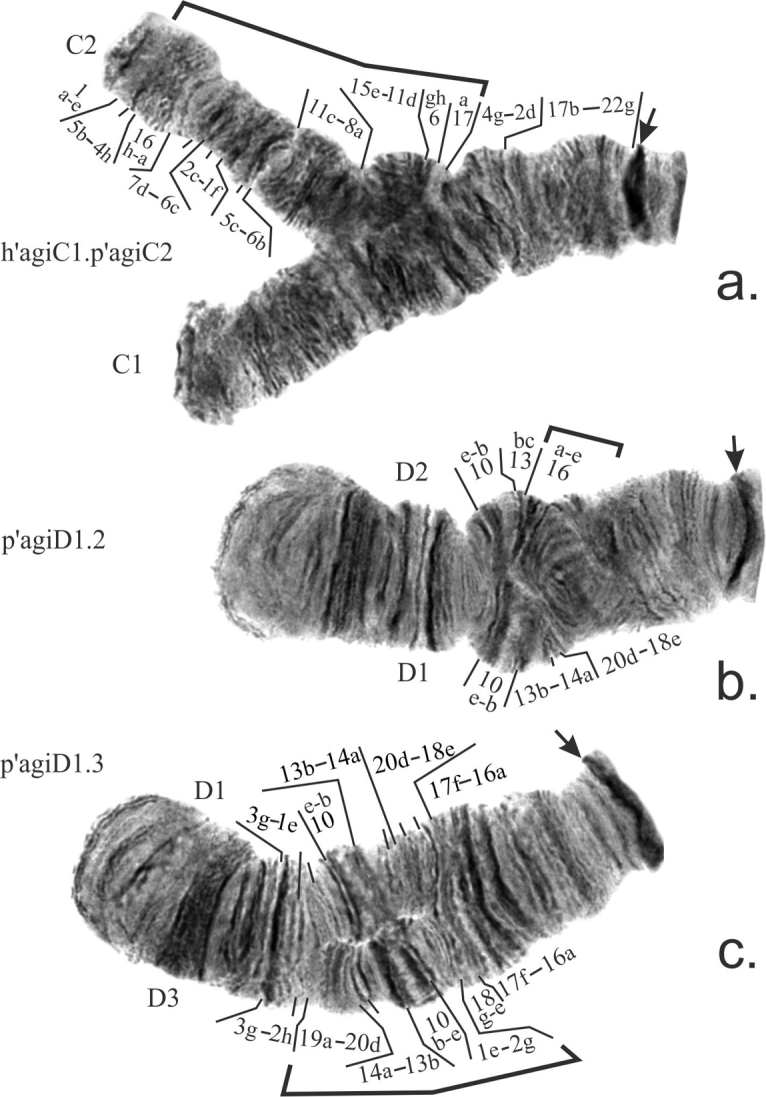
Inversions in arms C (**a**) and D (**b, c**) found in populations of *Ch.agilis.* Designations as on Fig. 3.

**Arm C** was monomorphic in all populations with the exception of the population from Travianoe Lake where the banding sequence p’agiC2 was found in a heterozygous state in a single larvae (Table 2, 3). It differs from the main banding sequence p’agiC1 by the large complex paracentric inversion. It was first described by Kiknadze and coauthors (2016), but it was not mapped in that study. We present its mapping for the first time. The banding sequence is identical to the main banding sequence of the sibling species Ch. sp. prope agilis (*Ch.agilis* 2) (Fig. 4a).

p’agiC1 1a-2c 6c-f 7a-d 16a-17a 6hg 11d-15e 8a-11c 6b-2d 17b-22g C

p’agiC2 1a-e 5b-4h 16h-a 7d-6c 2c-1f 5c-6b 11c-8a 15e-11d 6gh 17a 4g-2d 17b-22g C

**Arm D** was also monomorphic in most populations, but in total has three banding sequences. Both p’agiD2 and p’agiD3 banding sequences differ from p’agiD1 by simple paracentric inversions in the middle of the arm (Fig. 4b, c). The banding sequence p’agiD2 is mapped for the first time.

p’agiD1 1a-d 4a-7g 18a-d 8a-10a 13a-11a 3g-1e 10e-b 13b-14a 20d-18e 17f-14b 21a-24g C

p’agiD2 1a-d 4a-7g 18a-d 8a-10a 13a-11a 3g-1e 10e-b 13bc 16a-17f 18e-20d 14a-13d 15e-14b 21a-24g C

p’agiD3 1a-d 4a-7g 18a-d 8a-10a 13a-11a 3g-2h 19a-20d 14a-13b 10b-e 1e-2g 18g-e 17f-14b 21a-24g C

The banding sequence p’agiD2 can be classified as rare as it was found in two populations from Siberia, while p’agiD3 at present should be considered unique as it was found in a single larvae in one population from the Far East (Table, 2, 3). The banding sequence p’agiD3 is new for the species and described for the first time.

**Figure 5.**
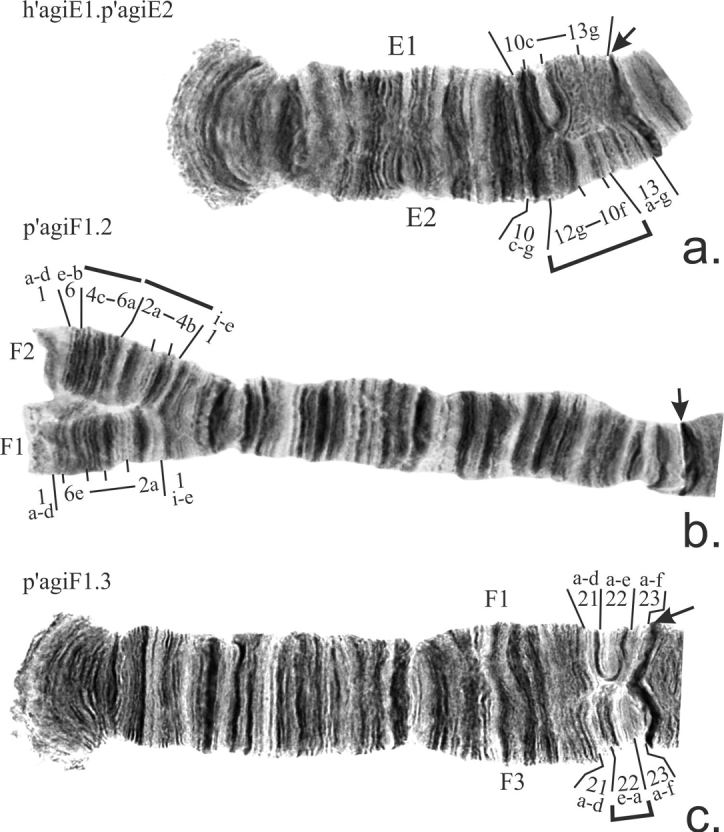
Inversions in arms E (**a**) and F (**b, c**) found in populations of *Ch.agilis.* Designations as on Fig. 3.

**Arm E** was monomorphic in all populations with the exception of the population from the pond on the Shadrikha river where the banding sequence p’agiE2 was found in a heterozygous state in a single larvae (Table 2, 3). It differs from the main banding sequence h’agiE1 by the simple short paracentric inversion located close to the centromeric region (Fig. 5a).

h’agiE1 1a-3e 5a-10b 4h-3f 10c-13g C (KV)

h’agiE1 1a-3a 4c-10b 3e-b 4b-3f 10c-13g C (GV)

p’agiE2 1a-3a 4c-10b 3e-b 4b-3f 10c-e 12g-10f 13a-g C (GV)

**Arm F** has three banding sequences. The main banding sequence p’agiF1 occurred in 100% of larvae from European and Far Eastern populations, and was dominant in all studied populations from Siberia (Table 2, 3). The banding sequence p’agiF2 was present in most Siberian populations, mostly in the heterozygous state, but homozygote p’agiF2.2 was also found (Table 3). It differs from p’agiF1 by the complex paracentric inversion on the distal part of the arm (Fig. 5b). The banding sequence p’agiF3 was found once in the pond on the Shadrikha river and differs from p’agiF1 by the very short paracentric inversion near the centromere (Fig. 5c). This sequence p’agiF3 is new for the species and described for the first time.

p’agiF1 1a-d 6e-1e 7a-10d 18c-a 11a-17d 18d-23f C

p’agiF2 1a-d 6e-b 4c-6a 2a-4b 1i-e 7a-10d 18c-a 11a-17d 18d-23f C

p’agiF3 1a-d 6e-1e 7a-10d 18c-a 11a-17d 18d-21d 22e-a 23a-f C

**Arm G** was monomorphic in all studied populations. It was not mapped as several active regions such as two nucleoli and two Balbiani Rings significantly increase the difficulty of comparison of banding patterns.

As was mentioned above, *Ch.agilis* was earlier considered as low polymorphic species, but our study of populations from Siberia had shown that at least in this region it has considerable level of chromosomal polymorphism, albeit not diverse with only couple inversions widespread in populations. Among studied populations of *Ch.agilis* the highest level of chromosomal polymorphism was found in Siberia (Novosibirsk and Altai regions) with 44.4–73.1% of heterozygotic larvae and 0.467–0.937 heterozygotic inversion per larvae, while European and Far Eastern populations were almost monomorphic (Table 2). The highest number of banding sequences – 12 – was found in the population from the pond on the Shadrikha river, while 9–10 banding sequences were present in most other populations.

All populations suitable for quantitative analysis (with number of specimens more than 10) were checked for deviations from Hardy-Weinberg equation. Only two populations – from ponds on the Shadrikha river (NSK-SH) and the Koynikha river (NSK-KO) – had shown a deviation from expected equation. In both populations number of heterozygotes in arm B exceeded expected values (P>0.99 and P>0.95, respectively): observed and expected frequencies of p’agiB1.2 were 49.8% and 41.1%, respectively, in NSK-SH, and 65.6% and 46.0% in NSK-KO. Unfortunately, the date on water quality in studied populations are not available so it is impossible to speculate about the cause of these deviations.

**Figure 6.**
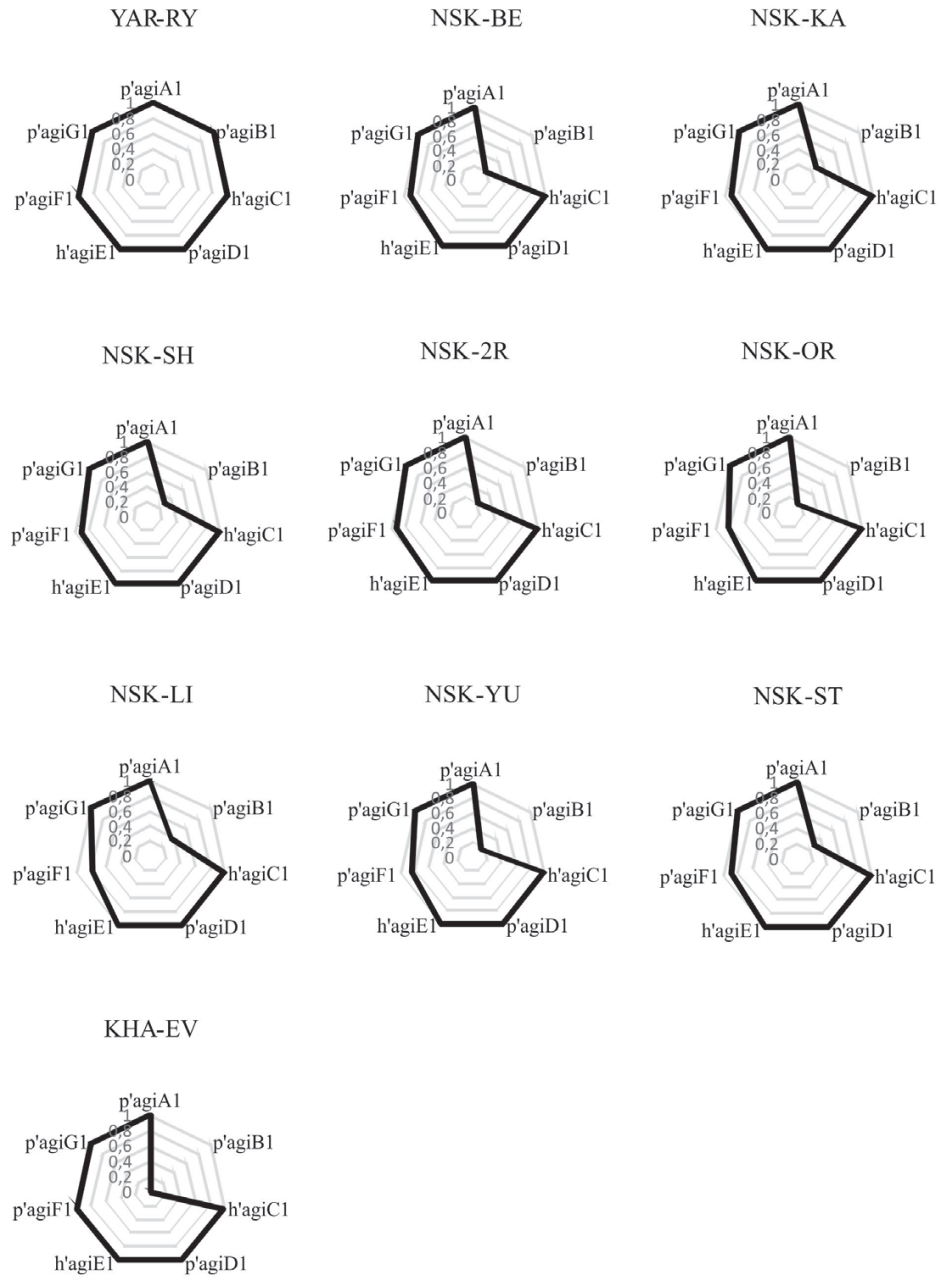
Cytogenetic structure (frequency polygons) of studied populations of *Ch.agilis*; YAR-RY, NSK-BE etc. – collections sites (see Table 1), p’agiA1, p’agiB1 etc. – main banding sequences of the species, 0.2, 0.4 etc. – frequencies of main banding sequences.

The cytogenetic structure of studied populations is shown on Fig. 6. The designation of types of population’s cytogenetic structure is done according to the work of Gunderina et al. (1999). As can be seen, population from Rybinskoe Reservoir belongs to cytogenetic type 0 (all main banding sequences are dominant), while all populations from Siberia and the Far East belong to type B (an alternative banding sequence is dominant in the arm B, in this case it is p’agiB2).

**Figure 7.**
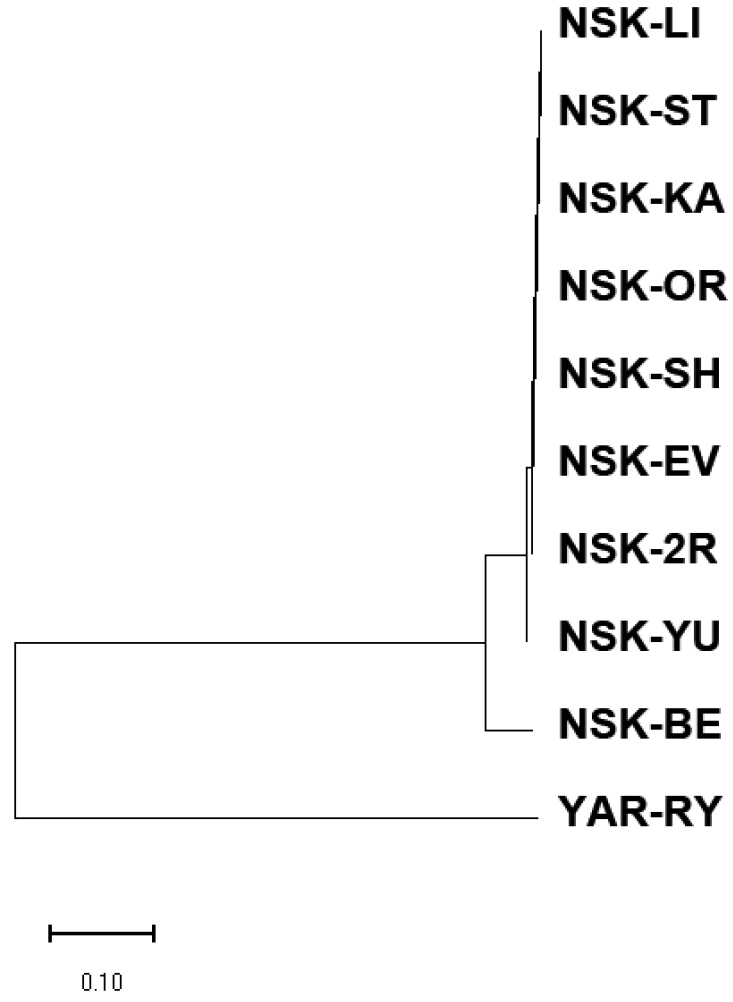
Phylogenetic tree of studied populations of *Ch.agilis*, calculated based on the neighbor-joining method.

The cytogenetic distances between populations varied from 0 to 0.155 (Table 4). They are well below the threshold of interspecies values, which for chironomids is considered to be around 1 for clearly differentiated species (Gunderina 2001). As expected, the minimal distances were observed between populations from Western Siberia, and the largest distance was seen between border populations – from Rybinskoe Reservoir and Evoron Lake. Phylogenetic tree calculated based on the neighbor-joining method is presented on Fig. 7.

**Table 4.** Cytogenetic distances between populations of *Ch.agilis*, calculated based on the Nei criteria (Nei 1972).

	YAR-RY	NSK-BE	NSK-KA	NSK-SH	NSK-2R	NSK-LI	NSK-OR	NSK-YU	NSK-ST
NSK-BE	0.106								
NSK-KA	0.081	0.002							
NSK-SH	0.078	0.002	0.000						
NSK-2R	0.096	0.000	0.001	0.001					
NSK-LI	0.116	0.001	0.003	0.004	0.002				
NSK-OR	0.069	0.007	0.003	0.003	0.006	0.007			
NSK-YU	0.116	0.001	0.003	0.004	0.002	0.000	0.007		
NSK-ST	0.080	0.002	0.000	0.000	0.001	0.003	0.002	0.003	
KHA-EV	0.155	0.006	0.012	0.013	0.007	0.007	0.025	0.006	0.013

Besides inversion polymorphism, genomic polymorphism in a form of additional B-chromosomes was also observed in two populations from Siberia – in ponds on the Ora and the Shadrikha rivers (Table 2).

The species *Ch.agilis* shows moderate level of chromosomal polymorphism in comparison with other well-studied species from plumosus group – *Ch.balatonicus*, *Ch.plumosus* and *Ch.entis*. For example, an average percent of heterozygotes found in Palearctic populations of *Ch.plumosus* is 63.2% with 0.95 inversion per larvae (Golygina and Kiknadze 2001), while these values for *Ch.agilis* are 45.1% and 0.52. The number of banding sequences found in populations of *Ch.agilis* is also lower – 9.2 on average vs 12.5 for Palearctic *Ch.plumosus* (Golygina and Kiknadze 2001), 15.3 for Palearctic *Ch.entis* (Kiknadze ert al. 2000) and 14.3 for *Ch.balatonicus* (Golygina et al. 1996). Although it is possible that this characteristic will change if more populations of *Ch.agilis* from different regions will be studied.

If we consider the distribution of chromosomal polymorphism between chromosome arms, *Ch.agilis* shows pattern similar to *Ch.balatonicus* where only two arms show high level of polymorphism in most populations, while other arms are almost completely monomorphic. At the same time, chromosome arms most heavily affected by inversion polymorphism are different in these two species: A and D in *Ch.balatonicus*, but B and F in *Ch.agilis.* In other two species – *Ch.plumosus* and *Ch.entis* – inversions are found with high frequencies in most or all chromosome arms.

At present, it is too early to draw a final conclusion about the characteristics of chromosomal polymorphism of *Ch.agilis*, as still not enough karyological data are available, especially for Europe, Eastern Siberia and the Far East. However, it is possible to assume that Western Siberia is the center of the species range of *Ch.agilis* and populations here have higher level of chromosomal polymorphism, which is also more diverse than in populations from the borders of the species range.

## References

[B1] Dévai Gy Miskolczi M Wülker W (1989) Standartization of chromosome arms B, C and D in *Chironomus* (Diptera: Chironomidae). Acta Biologica Debrecina. Supplementum Oecologica Hungarica Fasc 2(1): 79–92.

[B2] Golygina VV Istomina AG Rakisheva AZh Kiknadze II (1996) New banding sequences in the *Chironomusbalatonicus* karyofund (banding sequence pool). Tsitologiya 38(8): 869–883. [In Russian].

[B3] Golygina VV Kiknadze II (2001) Karyofund (banding species pool) of *Chironomusplumosus* (Diptera, Chironomidae) in Palearctic. Tsitologiya 43(5): 507–519. [In Russian] 11517668

[B4] Golygina VV Kiknadze II (2008) The revision of chromosome I (AB) mapping in *Chironomusplumosus* group (Diptera: Chironomidae). Comparative Cytogenetics 2(1): 37–55. 10.3897/CompCytogen.v12i2.23327 PMC5999685 29904571

[B5] Golygina VV Kiknadze II (2012) A revision of chromosome II (CD) mapping in *Chironomusplumosus* (Linnaeus, 1758) group (Diptera, Chironomidae). Comparative Cytogenetics 6(3): 249–266. 10.3897/compcytogen.v6i3.2831 24260666 PMC3833799

[B6] Golygina VV Kiknadze II (2018) A revision of chromosome III (EF) mapping in *Chironomusplumosus* (Linnaeus, 1758) group (Diptera, Chironomidae). Comparative Cytogenetics 12(2): 201–222. 10.3897/compcytogen.v12i2.23327 29904571 PMC5999685

[B7] Gunderina LI Kiknadze II Golygina VV (1999) Intraspecific differentiation of the cytogenetic structure in natural populations of *Chironomusplumosus* L., the central species in the group of sibling species (Chironomidae: Diptera). Russian Journal of Genetics 35(2): 142–150.

[B8] Gunderina LI (2001) Genetic variation in evolution of chironomids (Diptera, Chironomidae). Dissertation of Doctor of Science, Novosibirsk, Russian Federation: Institute of Cytology and Genetics SB RAS, 360 pp. [In Russian]

[B9] Keyl H-G (1962) Chromosomenevolution bei *Chironomus*. II. Chromosomenumbauten und phy­logenetische Beziehungen der Arten. Chromosoma 13(4): 464–514. 10.1007/BF00327342

[B10] Keyl H-G Keyl I (1959) Die cytologische Diagnostik der Chironomiden. I. Bestimmungstabelle für die Gattung *Chironomus* auf Grund der Speicheldrüsen-Chromosomen. Archiv für Hydrobiologie 56(1/2): 43–57.

[B11] Kerkis IE Kiknadze II Istomina AG (1989) Comparative analysis of karyotypes of three sibling-species in plumosus group (Diptera. Chironomidae). Tsitologiya 31: 713–720. [In Russian]

[B12] Kiknadze II Shilova AI Kerkis IE Shobanov NA Zelentzov NI Grebenjuk LP Istomina AG Prasolov VA (1991) Karyotypes and morphology of larvae in the tribe *Chironomini*. Novosibirsk, 113 pp. [In Russian]

[B13] Kiknadze II Butler MG Golygina VV Martin J Wülker WF Sublette JE Sublette MF (2000) Intercontinental karyotypic differentiation of *Chironomusentis* Shobanov, a Holarctic member of the *C.plumosus* group (Diptera, Chironomidae). Genome 43(5): 857–873. 10.1139/g00-052 11081977

[B14] Kiknadze II Istomina AV Golygina VV Gunderina LI (2016) Karyotypes of Palearctic and Hol­arctic species of the genus *Chironomus* [Electronic resource]. Russian Academy of Sciences, Siberian Branch, Federal Research Center Institute of Cytology and Genetics. Novosibirsk: Academic Pub­lishing House “GEO”, 489 pp. http://elibrary.ru/item.asp?id=27246690

[B15] Krastanov BD Michailova PV (2008) Cytotaxonomic Characteristic of Four Species of plumosus Group in Genus *Chironomus* Meigen 1803 (Diptera: Chironomidae) from Bulgaria and Poland. Acta Zoologica Bulgarica, Suppl. 2: 49–60.

[B16] Maximova FL (1976) The karyotype of *Chironomusplumosus* from Ust’-Izhora wild population of Leningrad region. Tsitologiya 18: 1264–1268. [In Russian]

[B17] Michailova P Krastanov B Kownacki A (2002) Cytotaxonomical characteristics of genus *Chironomus* Meigen (Diptera: Chironomidae) from different localities of Poland. Annales Zoologici 52(2): 215–225.

[B18] Nei M (1972) The genetic distance between populations. American Naturalist 106: 283–292. 10.1086/282771

[B19] Plokhinsky NA (1967) Algorithms of biometry. Moscow University Press, 82 pp.

[B20] Shobanov NA (1994) Karyofund of *Chironomusplumosus* (L.) (Diptera, Chironomidae). I. Standartization of bands according to the Maximova system. Tsitologiya 36(1): 117–122. [In Russian]

[B21] Shobanov NA Djomin SYu (1988) *Chironomusagilis* – new species of group plumosus (Diptera, Chironomidae). Zoologicheskiy Zhurnal 67(10): 1489–1497. [In Russian]

